# Abusive leadership: A moderated-mediation through leader-member exchange and by organizational politics

**DOI:** 10.3389/fpsyg.2022.983199

**Published:** 2022-11-10

**Authors:** Amos Drory, Or Shkoler, Aharon Tziner

**Affiliations:** ^1^Peres Academic Center, Rehovot, Israel; ^2^École des Hautes Études Commerciales de Montréal, Université de Montréal, Montreal, QC, Canada; ^3^Netanya Academic College, Netanya, Israel; ^4^Tel-Hai College, Qiryat Shemona, Israel

**Keywords:** abusive leadership, organizational politics, leader-member exchange, organizational citizenship behaviors, counterproductive work behaviors, turnover intentions, moderated-mediation

## Abstract

Abusive leadership has been shown to have adverse consequences for both the employees and the organization. In the current paper, the impacts of such a leadership style on workers’ turnover intentions (TIs), counterproductive work behaviors (CWBs) and organizational citizenship behaviors (OCBs) are investigated through a dyadic lens—the mediation of leader-member exchange (LMX). Furthermore, when the workplace atmosphere is also tainted by high level of perceived organizational politics (POP) (as a moderator), these relationships deepen and and/or change (for the worse). To test the moderated-mediation research model, an online sample of 619 participants was obtained. The results support an interesting moderated-mediation of LMX by POP. Theoretical and practical implications, limitations and future research suggestions are discussed.

## Introduction

The literature often emphasizes the positive aspects of leadership in organizations—especially with the rise of positive psychology—but, in tandem, puts less emphasis on the darker sides of the managerial spheres at workplaces (Naseer et al., 2016). There is, however, increasing awareness that workplace abuse is a severe and widespread social phenomenon, which invades all forms of work environments, and encompasses many behaviors at varying degrees of intensity and negativity (Aharoni-Goldberg et al., 2019). Not only is the phenomenon prevalent, it is also consistently linked to detrimental consequences for both the employees and the organization; for example, feelings of shame, turnover intentions (TIs), fear, employee silence, deviant behaviors, impaired performance, reduced creativity, and more (e.g., Naseer et al., 2016; Xu et al., 2020; Jain et al., 2021; Korman et al., 2021; Liao et al., 2021; Bokek-Cohen et al., 2022). As such, it is clear that there is a prime necessity to scrutinize and research this side of managers, in the aim of creating more noise and buzz surrounding abusive leadership in order to foster discussion at the organizational and public levels (for both academics and practitioners). In the current paper, we investigate the effects of abusive leadership and leader–member exchange (LMX) on employees’ attitudes. We further examine the role of perceived organizational politics (POP) (e.g., Lam and Xu, 2019) in moderating the above relationship. Additionally, while some of the links discussed in the paper have been investigated previously (e.g., Xu et al., 2020), none, to the best of our knowledge, have integrated them into a single coherent model, as is illustrated and elaborated in the next sections.

## Theoretical background

### Organizational theories

The current research draws from several important organizational theories, namely: (1) social exchange theory (SET; Blau, 1964), (2) reciprocity theory (Gouldner, 1960), (3) equity theory (Adams, 1965), and (4) expectancy theory (Vroom, 1964; Oliver, 1974). These have served as a guide and road signs for the theory building and hypotheses.

*Social exchange* and *reciprocity* theories argue that social relationships are based on the trust that gestures of goodwill will be reciprocated, and vice versa (i.e., an eye for an eye) (e.g., Martin et al., 2016; Shkoler and Tziner, 2017). A behavior producing positive outcomes will be repeated, and one that elicits negative consequences will be inhibited by the individual (Homans, 1958). In this article, we concentrate on two important exchange foci in the organization—employee–supervisor and employee–organization (Cole et al., 2002)—as we aim to identify how abusive behavior can affect organizational outcomes through its impact on workers. In addition to these, *equity theory* propounds that when employees nurture perceptions of injustice (for example), they act to rectify the situation in an attempt to create balance. Such behaviors include reducing their inputs in order to rectify their perceived input–outcome imbalance (e.g., Greenberg and Scott, 1996), developing negative feelings toward the organization, experiencing reduced motivation, manifesting distrust (toward the workplace and/or the manager), and even acting against the organization (e.g., Daileyl and Kirk, 1992; Skarlicki and Folger, 1997). Finally, one of the main premises of *expectancy theory* stresses that employees will behave/act in a specific manner because they are motivated to select a specific behavior (over other alternatives), nested in what they expect the result of that selected behavior will be. Importantly, however, the outcome is not the only factor molding the individual’s motivation to select a certain behavior (Vroom, 1964; Oliver, 1974). As such, for example, if a worker expects abusive behavior from their supervisor, they will choose not to speak up or share information with the manager (i.e., employee silence) (e.g., Xu et al., 2020; Jain et al., 2021).

In the next section, the study’s constructs are defined, elaborated and linked, capitalizing on the above-mentioned theories.

### Constructs of the study

#### Abusive leadership

Abusive leadership is defined as subordinates’ perceptions of the extent to which their supervisors engage in sustained display of hostile verbal and non-verbal behaviors, excluding physical contact (Tepper, 2000, p. 178). This may include disrespect, aggression, mistreatment, verbal abuse, emotional abuse, humiliation, degradation, anger tantrums, ridiculing, belittling, and more (e.g., Shore et al., 2011; Rice et al., 2020; Liao et al., 2021). The abusive manager is able to exert this kind of sway over the worker due to the simple fact of their authority as a superior, while the employee is left to comply with the whims of said supervisor (Rice et al., 2020). This demoralizing experience for workers inevitably taxes their psyche, impairs their self-efficacy, and drains them of personal strength and resources, inducing stress (Hobfoll, 1989, 2011; Duffy et al., 2002; Mawritz et al., 2014), and increasing perceived unfairness (e.g., Lind and Tyler, 1988; Tyler and Lind, 1992; Cropanzano et al., 2001). Eventually, this might diminish their feeling of inclusivity and belonging at the workplace (see Rice et al., 2020), escalating a sense of insecurity, shame, anticipation of social exclusion, job dissatisfaction, and job neglect. It can compromise manager–employee relations (Martinko et al., 2013; Palanski et al., 2014; Korman et al., 2021; McLarty et al., 2021), while promoting withdrawal from resource-consuming activities (e.g., information sharing or speaking up) (Kish-Gephart et al., 2009; Ng and Feldman, 2012; Xu et al., 2020). Salton Meyer and Ein-Dor (2021) found that the links between abusive supervision, burnout and lower job satisfaction were highly meaningful as they indicated substantial adverse psychological influences on employees’ daily feelings and experiences at work, thus negatively impacting their work-related effectiveness and productivity. In a meta-analysis and empirical review on abusive supervision, Mackey et al. (2017) indicated that abusive supervision is an organizational occurrence that has significant academic and practical consequences.

#### Leader–member exchange

As mentioned, abusive supervision inescapably impacts the relationships between a worker and their immediate manager (assuming the latter is the abuser). The dyadic supervisor–supervised relations are known as LMX. This is based on the observation that in dyadic relationships and organizational settings, managers tend to develop and use different relationship and management styles with each of their subordinates with varying outcomes (Dansereau et al., 1975; Graen and Cashman, 1975; Matta and Van Dyne, 2020). Different styles also produce different attitudes in the subordinates themselves (Ilies et al., 2007; see also Shkoler and Tziner, 2020; Tziner et al., 2020). Capitalizing upon social exchange theory (SET; Blau, 1964) and reciprocity theory (Gouldner, 1960), employees in good relationships with their managers (i.e., high LMX) usually feel obliged to mutually reciprocate (see also Adams, 1965). As such, high-quality LMX results in high levels of mutual trust, respect, organizational identification, knowledge sharing, felt (mutual) obligation, support, and commitment from leaders to subordinates, and vice versa. It is important to note that bad relations (i.e., low LMX) with a manager tend to result in reciprocal bad (negative) behavior, and accordingly may eventually lead to work misbehaviors (Ilies et al., 2007; Breevaart et al., 2015; Lebrón et al., 2018; Lee et al., 2019; Shkoler et al., 2019; Teng et al., 2020; Newton and Perlow, 2021).

#### Abusive leadership and leader–member exchange

The link between abusive leadership and LMX is fairly straightforward—abuse will be met with distrust and antagonism. That is to say, the more abusive the leader, the worse their relationship with the abused employee. Also, subordinates generally evaluate supervisors who exhibit high degrees of disagreement with their own colleagues as more abusive, especially when subordinates have a low-quality LMX relationship with the supervisor (Harris et al., 2011). This happens because of the reciprocity in the exchanges between the two parties, nested in balanced workplace equity (Gouldner, 1960; Blau, 1964; Adams, 1965). Specifically, there are three main commitments for social exchanges—namely, the commitment to: (1) give; (2) receive; and (3) reciprocate (Mauss, 1967). Once an assumption of goodwill intentions, for example, has been violated, this might induce dissonance and shock to the individual’s system (Festinger, 1954, 1957). As consequence and *ad hoc* solution (i.e., coping mechanism), employees (as targets of abusive management) might develop negative attitudes toward the supervisor and/or the organization, blaming them for the abuse (Bowling and Michel, 2011; Shoss et al., 2013; Lian et al., 2014; Tröster and Van Quaquebeke, 2021), and subsequently adjusting their behaviors toward the same entities (e.g., Festinger, 1954, 1957; Adams, 1965). As such, we hypothesized the following:

*H1: Abusive leadership is negatively associated with leader–member exchange so that an increase in abusive leadership is followed by a decrease in LMX.*

#### Organizational citizenship behaviors

Tziner et al. (2020) have summarized: Organizational citizenship behaviors (OCBs) are voluntary prosocial behaviors toward the organization or its members, which have a positive impact on effectiveness and efficiency. OCBs are typically seen as outside the formal job description, spontaneous and voluntary behaviors, not apparently or explicitly rewarded, and positive in terms of the organization or group enjoying the behavior (Organ, 2006). Such behaviors include helping others with their workload or problem solving, preventing intra-work discord, and working beyond what is required by organizational norms (Organ, 1988; Tziner et al., 2020, p. 4-5; references are from the original text).

Evidently, various job experiences can foster (or inhibit) such extra-role behaviors, particularly the interactions with the supervisor (e.g., Zhang et al., 2018; Rice et al., 2020; Teng et al., 2020; Tziner et al., 2020). As was previously mentioned, when a supervisor is supportive (e.g., trust, emotional support, information sharing), employees feel obliged to reciprocate, providing mutual benefit for both sides, and this might drive them to exhibit OCBs (Gouldner, 1960; Blau, 1964; Tziner et al., 2020).

#### Counterproductive work behaviors

As opposed to OCBs, counterproductive work behaviors (CWBs) are any intentional behaviors on the part of an organizational member viewed by the organization as contrary to its legitimate interests (Sackett and DeVore, 2001, p. 145; see also Cohen-Charash and Mueller, 2007; Ho, 2012). CWBs have considerable economic, sociological, and psychological implications for organizations, as they often violate important organizational norms, and harms organizations in many ways; for example, their goals, employees, procedures, productivity, and profitability (Spector et al., 2006; Aubé et al., 2009; Vardi and Weitz, 2016). Examples of CWBs are speaking ill of other workers, harassment, insulting and/or ignoring others at work, theft, intentional sabotage, and more (Robinson and Bennett, 1995; Bennett and Robinson, 2000; Gruys and Sackett, 2003; Spector et al., 2006, 2010). In this regard, the role of the managers is pivotal, as they can foster positive and/or negative attitudes and experiences for their employees, as previously discussed. Indeed, CWBs can stem from work social exchanges with coworkers and supervisors alike, work experiences and stressors, emotional/cognitive states, and motivational aspirations (Chen and Spector, 1992; Mitchell and Ambrose, 2007; Shkoler and Tziner, 2017; Lebrón et al., 2018; Zhang et al., 2018; Shkoler et al., 2019, 2021; Chen et al., 2020). In this instance, in addition, the intimate and crucial role of the supervisor becomes apparent. They can either promote positive experiences (for employees) at work or, alternatively, elicit negative attitudes, each with difference outcomes. However, adverse experiences may lead workers to react against the manager and/or the organization (e.g., Adams, 1965; Kelloway et al., 2010).

#### Turnover intentions

Voluntary employee turnover (i.e., intention and actually leaving the organization) has long been a concern for managers due to the significant costs associated with it across multiple organizational performance dimensions (Hom et al., 2012). Among others, these include time, effort, and financial resources necessary to recruit, select, and train new employees to replace those who have left the workplace. This is in addition to the potential damage to organizational reputation since turnover signals an unattractive place to work (e.g., Hom et al., 2017; Xu et al., 2020). Other concerns are the lowered morale and satisfaction of those remaining in the organization, in addition to the lowered productivity in teams whose members have left (Hom et al., 2017; Lee et al., 2018; Steffens et al., 2018).

There is extant research noting these costs, and, in fact, research on turnover is fairly exhaustive and goes back several decades (e.g., Griffeth et al., 2000; Park and Shaw, 2013). In their review of 100 years of turnover research Hom et al. (2017) called for future studies to better capture differences in context and to recognize that contextual factors can shape the influence of turnover antecedents (p. 540). Managers (specifically, with regard to abusive leadership and LMX) are one such contextual factor (e.g., Rice et al., 2020; Korman et al., 2021; Thompson et al., 2021).

#### Abusive leadership and organizational citizenship behaviors, counterproductive work behaviors and turnover intentions

Abuse of any kind is detrimental and, in the context of work, can have ramifications for the employees and the organization alike. As mentioned, at a basic level, the manager can foster a good working climate, just as they can mandate an abusive one. However, this does not occur in a vacuum, and the employees react in a reciprocal fashion; on the one hand, goodwill may be met with mutually good intentions and support, but on the other hand, abuse might be met with resistance/retribution, and a plethora of negative attitudes (Gouldner, 1960; Blau, 1964; Adams, 1965). Abusive leadership delivers a shock to the employees’ system (e.g., unfolding turnover model; Lee and Mitchell, 1994) and, as a consequence, evokes antagonism and retaliation (e.g., CWBs), reconsideration of current work (e.g., TIs), and can lead to reduced performance and extra-role behaviors (e.g., OCBs). Subordinates’ perceptions of abusive supervision were found significantly associated with their greater intention to quit, which is regarded a predictor of actual resigning (Shapira-Lishchinsky, 2005). Consequently, we hypothesized the following:

*H2: Abusive leadership is negatively associated with organizational citizenship behaviors so that an increase in abusive leadership is followed by a decrease in OCB.*

*H3: Abusive leadership is positively associated with counterproductive work behaviors so that an increase in abusive leadership is followed by an increase in CWB.*

*H4: Abusive leadership is positively associated with turnover intentions so that an increase in abusive leadership is followed by an increase in TI.*

#### Leader–member exchange as a mediational mechanism

LMX’s role as a potential mediator (e.g., Sharif and Scandura, 2017; Tziner et al., 2020) is highlighted and strongly connected to abusive leadership. Although causal links cannot be inferred, an abusive management style implicates the relationships between the supervisor and the employee, and only in rarer cases does it work the other way around (i.e., that low-quality exchanges lead to abuse). As such, abusive leadership is the progenitor of varying levels of leader–member exchanges, such that the former predicts the latter. It should be noted that although abusive leadership and LMX are related, it would be inconceivable to expect high levels of abuse to coexist with high LMX. High levels of leadership abuse would suppress the potential for high LMX, yet some variance in LMX at lower levels is still possible as LMX is determined by additional factors beyond leadership abuse. Furthermore, the two concepts are firmly intertwined because the supervisor is, at the same time, both abuser and manager, and in this sense the effects of abusive leadership on OCB, CWB, and TI can be both direct and indirect (through a decline in LMX quality). If so, LMX becomes a mediator between abusive leadership and its outcomes (OCB, CWB, and TI). It is important to note, however, that this mediation is not total, as abuse cannot be ignored or turned a blind eye to. That is to say, abusive leadership will invariably have a direct impact on its outcomes, but we argue that it will also indirectly affect them as well (through LMX). As such, we hypothesize the following:

*H5: Leader–member exchange partially mediates the associations between abusive leadership and its outcomes (OCB, CWB, and TI).*

#### Perceived organizational politics—A moderated-mediation

Capitalizing on Hom et al.’s (2017) call for the exploration of contextual factors related to TI, we expand its scope and boundaries to the other variables in our research. The leading question is whether there is a contextual factor that might exacerbate or ease the negative impact of abusive behavior or interact with the managerial style of the supervisor. The factor we applied in this study is POP.

The academic interest and study of politics in organizations has been growing steadily over the last four decades. The definition of political behavior in organizations typically includes characteristics such as discretionary behavior, self-serving behavior, influence tactics, and often, being harmful to other individuals and to organizational goals (Porter et al., 1981; Drory and Romm, 1990).

The study of politics in organizations during the last two decades has generally fallen into one of two categories, viewed, at least implicitly, as largely independent. One category focuses on the nature of actual political behavior, types of tactics and strategies, and their consequences. The other category is concerned with the *perceptions* of politics in work environments by individual employees, the antecedents of such perceptions, and their consequences.

The present study focuses entirely on the subjective category of politics in organizations, perceived organizational politics—namely, POP.

POP refers to individuals’ subjective perceptions of others’ political activities (not their own), such as favoritism, suppression of competing entities, and the manipulation of organizational policies (Kacmar and Ferris, 1991, p. 203; see also Ferris et al., 2002). Naseer et al. (2020) emphasized that the perceptions of politics are an important characteristic of the work environment. The role and impact of POP has been extensively investigated by looking at a wide variety of related variables from organizational attitudes and work-related behavior, personality variables, stress and strain, cultural variables and leadership-related variables (Vigoda-Gadot and Drory, 2016). While there are studies focusing on the relationship between POP and leadership style (for example, Durrani, 2014), no empirical findings pertaining to the association between POP and abusive leadership are available to the best of our knowledge.

In the current study, the role of POP is examined as a moderator-mediator of the relationship between *abusive leadership-through-LMX on the outcomes (OCB, CWB, and TI).*

While the majority of empirical studies to date treated POP as an independent or dependent variable, there are some cases in which it is used as a moderator or mediator. One interesting study (Hochwarter et al., 1999) used POP as a moderator, examining its moderating influence on the relationship between conscientiousness and job performance. Replicating previous findings (e.g., Barrick and Mount, 1993), conscientiousness was found to have a significant positive relationship with supervisor ratings of performance. Some other studies applied POP as a mediating variable. Vigoda (2000), for example, found politics perceptions to be “a good mediator between constructs of job congruence and employee performance” (p. 202). Other studies also demonstrated the potential of POP as a mediator (Ferris et al., 1996; Valle and Perrewé, 2000).

The hypothesis pertaining to POP as a moderator of the relations between abusive leadership and work-related behavior is based on the distinction between the two major variables in this study—namely, POP and abusive leadership. Superficially, the two variables represent negative and undesirable factors from the employee’s point of view. Both variables assess the employee’s subjective perception, yet they actually measure perceptions that are different in a very meaningful way.

To illustrate, the measure of leadership abuse consists of items that directly focus on the dyadic relationship between the respondent and their superior, and describes concrete examples of the superior’s actions directed at the respondent. A sample item is: “Puts me down in front of others, tells me I am incompetent, or is rude to me.”

The measure of POP, on the other hand, focuses on the organizational climate domain. It deals with a more general feeling of injustice and unfairness, not pointing a finger at any individual. For example: “Favoritism rather than merit determines who gets ahead around here,” “People here usually don’t speak up for fear of retaliation by others.” We can therefore make a distinction between two separable domains. One is the superior–subordinate dyadic domain and the other is the climatic domain.

In this study we propose that POP, when high, may have a masking or suppressive effect on the role of the superior–subordinate dyadic relations in affecting employee intentions and behavior toward the organization. In other words, high POP, being a more fundamental and long-term factor affecting the individual’s future wellbeing, may be a greater source of concern than the individual superior’s behavior or the quality of the relations between them. It may create bitterness and negative feelings toward the organization, well beyond the concern with one’s individual superior. Under such conditions, it is suggested that the impact of the superior–subordinate domain in shaping a subordinate’s attitudes and behavior toward the organization is reduced as the subordinate is more worried and more influenced by the negative climate effect.

**FIGURE 1:**
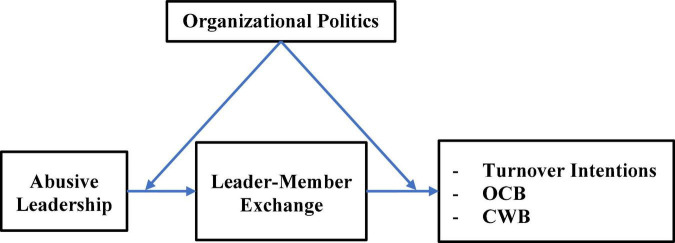
Research model. OCB, organizational citizenship behavior; CWB, counterproductive work behavior.

Hence, the mediational impact of abusive leadership on organizational outcomes, through LMX, is conditioned (moderated) by the level of the POP perceived by the employee. As such, we hypothesize the following (see Figure 1 for the overall research model):

*H6: Organizational politics perceptions (POP) moderate the mediational mechanism of abusive leadership-through-LMX on the outcomes (OCB, CWB, and TI).*

## Materials and methods

### Participants and procedure

#### Sample size calculation

G*Power (v. 3.1.9.7) statistical software was utilized to determine a minimal sample size for the analyses. By using a standard α error probability of 5%, power of 95% and a fixed effect size of.15 for 3*3 predictors*outcomes, the *minimal a priori* sample size is *n* = 166 (and *n* = 245 for effect size of 0.10). Another method to determine the sample size is the rule of thumb of, at least, 30 observations per variable (invoking the central limit theorem), in which case 6 variables in total reflects a total minimal sample size of *N* = (6*30) = 180 required so that the data will also converge and approximate a standard normal distribution. Based on these *a priori* analyses, any sample size above 245 (as the stricter upper bound) is considered more than adequate for the current research (Islam et al., 2022; see also Islam and Hussain, 2022).

Demographically, the current sample consisted of 619 participants, 50.2% of which are males and 49.8% females, between the ages of 19 and 67 years (*M* = 38.47, *SD* = 10.84). Almost half of them were single (48.8%), 42.8% were married, and 8.4% were divorced. Most of the respondents were Christian (91.6%), 5.2% were Jewish, and 3.2% were Muslim.

#### Data collection procedure

The research survey was uploaded the Prolific online platform,^1^ through which the sample was gathered.

### Measures

*Abusive leadership* was gauged using Tepper’s (2000) abusive supervision questionnaire consisting of 12 items on a Likert-scale between 1 (I cannot remember him/her ever using this behavior with me) and 6 (He/she uses this behavior very often with me); e.g., “My boss/supervisor puts me down in front of others.” Reliability coefficient (Cronbach’s alpha), mean and standard deviation of this variable is presented in Table 1.

**TABLE 1:** Zero-order Pearson correlation matrix (*N* = 619).

	*M*	*SD*	1	2	3	4	5	6	7	8	9
Gender	1.50	-									
Married	0.43	-	–0.03								
Age	38.47	10.84	–0.07	0.24***							
Abusive Leader	1.53	0.83	–0.04	0.01	0.01	**(0.96)**					
Org. Politics	3.26	0.95	0.10*	0.03	0.03	0.46***	**(0.88)**				
LMX	3.41	0.86	–0.04	–0.01	–0.01	–0.55***	–0.58***	**(0.92)**			
CWB	1.59	0.53	–0.03	–0.11**	–0.11**	0.45***	0.33***	–0.31***	**(0.86)**		
OCB	4.06	0.67	–0.02	0.11**	0.11**	–0.06	–0.16***	0.28***	–0.37***	**(0.79)**	
Turnover	3.13	1.56	–0.02	–0.10*	–0.10*	0.29***	0.43***	-0.45***	0.34***	–0.27***	**(0.91)**

*p < 0.05, **p < 0.01, ***p < 0.001. Cronbach’s Alpha coefficients are depicted in bold parenthesis on the diagonal. For dichotomous variables, only the mean is provided. Gender: 0, male; 1, female. Married (dummy-recoded): 0, not currently married; 1, currently married. Org., organizational; LMX, leader–member exchange; CWB, counterproductive work behavior; OCB, organizational citizenship behavior.

*Perceptions of organizational politics (POP)* were gauged using Kacmar and Carlson’s (1997) questionnaire consisting of 12 items on a Likert-scale between 1 (strongly disagree) and 6 (strongly agree); e.g., “Favoritism rather than merit determines who gets ahead around here.” Reliability coefficient (Cronbach’s alpha), mean, and standard deviation of this variable is presented in Table 1.

*Leader–member exchange (LMX)* was gauged using the LMX7 measure (Graen and Uhl-Bien, 1995) consisting of 7 items on a Likert-scale between 1 and 6; e.g., “How well does your leader understand your job problems and needs?” However, anchors of the scale are different for each item, while the *lowest* (1) are described as: rarely, not a bit, not at all, none, none, strongly disagree, and extremely ineffective (for items 1 through 7, respectively), the *highest* anchors (6) are presented as: very often, a great deal, mostly fully, very high, very high, strongly agree, and extremely effective (for items 1 through 7, respectively). Reliability coefficient (Cronbach’s alpha), mean, and standard deviation of this variable is presented in Table 1.

*CWBs* were gauged using the same instrument as used by Chernyak-Hai and Tziner (2014), consisting of 15 items on a Likert-scale between 1 (very rarely) and 6 (very often); e.g., “Purposely damaged a piece of equipment or property.” Reliability coefficient (Cronbach’s alpha), mean, and standard deviation of this variable is presented in Table 1.

*OCBs* were gauged using Williams and Anderson’s (1991) questionnaire consisting of 16 items on a Likert-scale between 1 (very rarely) and 6 (very often); e.g., “I assist the supervisor with his/her work.” Reliability coefficient (Cronbach’s alpha), mean, and standard deviation of this variables is presented in Table 1.

*Turnover intentions (TIs)* were gauged using Cammann et al.’s (1979) questionnaire consisting of 3 items on a Likert-scale between 1 (very unlikely) and 6 (very likely); e.g., “How frequently do you think about leaving your current organization?” Reliability coefficient (Cronbach’s alpha), mean, and standard deviation of this variables is presented in Table 1.

## Results

### Common-method bias analysis

Two methodologies were employed to test for the extent of possible common-method variance (CMV), accounting for variable intercorrelations in the results (see Podsakoff et al., 2003). The methods were: (1) Harman’s single-factor method (all items are loaded into one common/marker factor); and (2) a common latent factor (CLF) method (all items are loaded into both their expected factors and one latent common method factor). Based on Harman’s single-factor model, we notice that the results of the analysis accounted for only 17.99% of the explained variance (fit indices are suggested by, for example, Byrne, 2010; Shkoler and Kimura, 2020; Shkoler et al., 2021): χ^2^(3,315) = 9611.63, *p* = 0.000, χ^2^/df = 2.90, CFI = 0.68, NFI = 0.70, NNFI = 0.77, GFI = 0.69, SRMR = 0.13, RMSEA (90% CI) = 0.18 (0.10–0.25), *p-close* = 0.000. Further, the CLF alternative model produced 16.03% of the explained variance: χ^2^(3,170) = 9,174.13, *p* = 0.000, χ^2^/df = 2.89, CFI = 0.70, NNFI = 0.86, NFI = 0.79, GFI = 0.82, SRMR = 0.12, RMSEA (90% CI) = 0.15 (0.07–0.18), *p-close* = 0.000. While these findings do not exclude the possibility of same-source bias (CMV), following Podsakoff et al. (2003), we note that if the explained variance accounted for by the single-factor is less than 50% (i.e., *R*^2^ < 0.50)—in conjunction with a poor model fit for each analysis—then this is a firm indication that CMB is an improbable confound to our findings.

### Zero-order correlations

In order to assess the inter-relationships among the variables/sub-scales in the current study, a zero-order Pearson correlation matrix was calculated, as presented in Table 1. The results show a significant negative correlation between abusive leadership and LMX (*r* = -0.55, *p* < 0.001), thus supporting Hypothesis 1. Furthermore, significant correlations were found between abusive leadership and two of the three employee behavioral indicators—namely, CWB (*r* = 0.45, *p* < 0.001) and turnover intent (*r* = 0.29, *p* < 0.001). Hypotheses 4 and 5 were thus supported, while Hypothesis 3 regarding the effect of abusive leadership and OCB was not supported by the results.

### Moderated-mediation analysis

**FIGURE 2:**
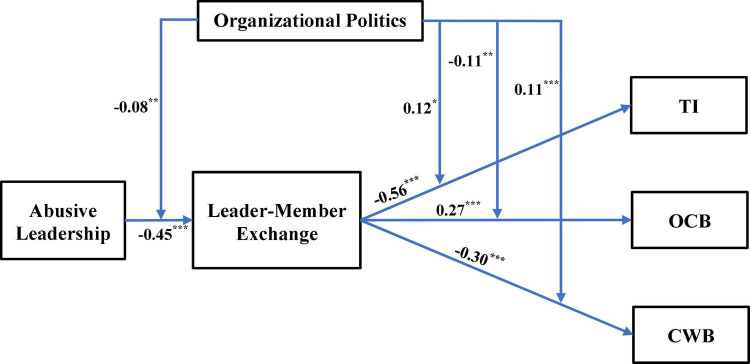
Path diagram and unstandardized regression coefficients. *p < 0.05, **p < 0.01, ***p < 0.001. TI, turnover intentions; OCB, organizational citizenship behavior; CWB, counterproductive work behavior.

**FIGURE 3:**
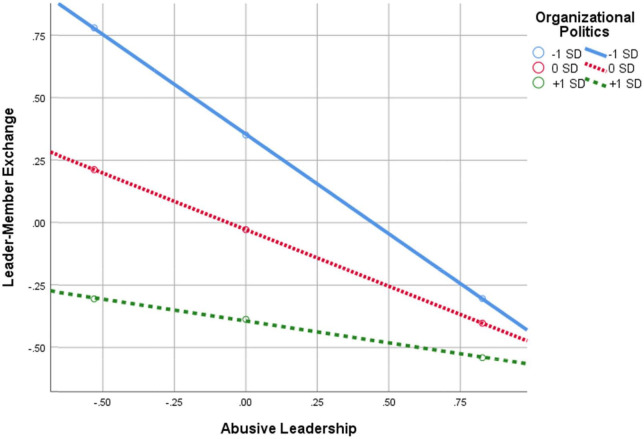
Interaction of abusive leadership × perception of organizational politics in predicting LMX.

**FIGURE 4:**
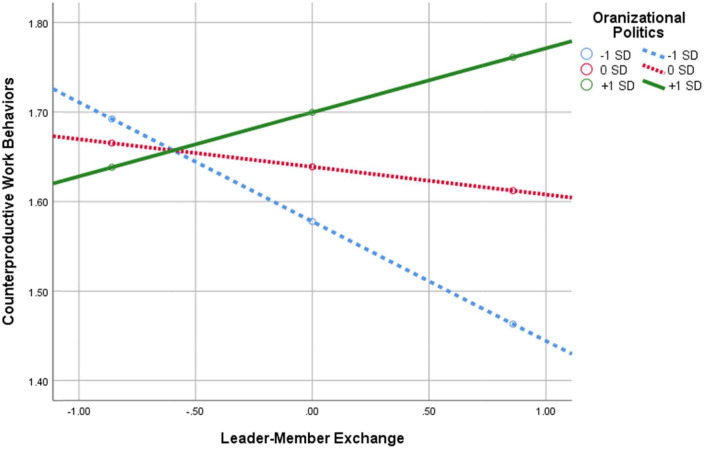
Interaction of leader–member exchange × perception of organizational politics in predicting CWB.

**FIGURE 5:**
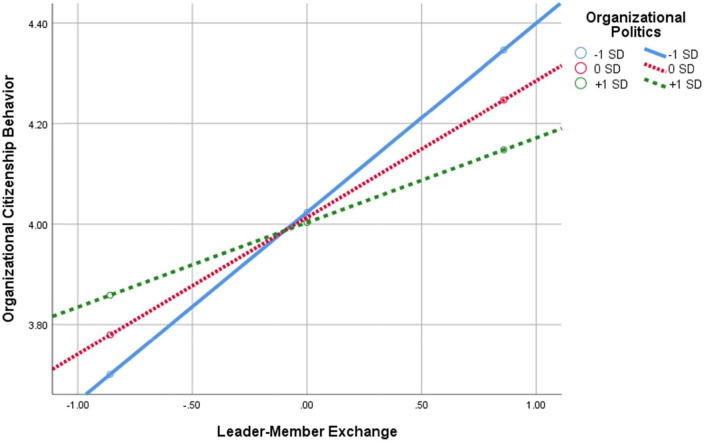
Interaction of leader–member exchange × perception of organizational politics in predicting OCB.

**FIGURE 6:**
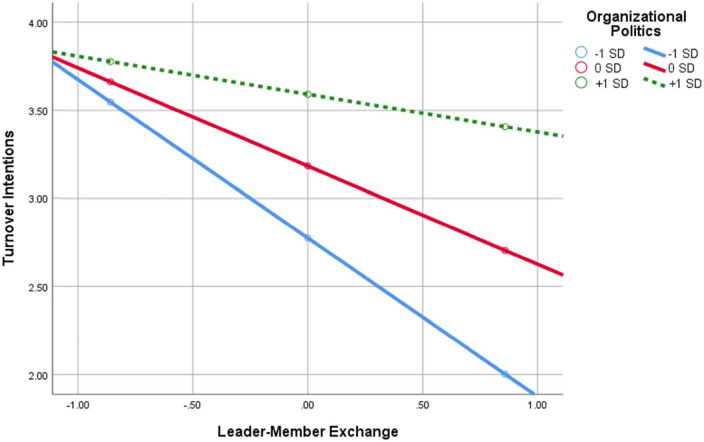
Interaction of leader–member exchange × perception of organizational politics in predicting turnover intentions.

In order to test the model (Figure 1), we first employed SEM analysis in AMOS (v. 24) to gauge the fit of the model: χ^2^(4) = 12.71, *p* = 0.013, χ^2^/df = 3.18, CFI = 0.99, NFI = 0.98, NNFI = 0.98, GFI = 0.97, SRMR = 0.02, RMSEA (90% CI) = 0.06 (0.03–0.10), *p-close* = 0.285. These indices suggest fit in the absolute sense, apart from the χ^2^/df ratio (see Byrne, 2010). Following this analysis, we utilized PROCESS macro (v. 3.5) in SPSS (v. 26) to test the moderated-mediation links (model #58) with 95% bias-corrected bootstrapping (5,000 resamples). To control for potential confounding effects, the following variables were considered as covariates: gender, age, religion and marital status. In addition, it is important to note that we used heteroscedasticity-consistent standard error (SE) estimators, as suggested by Hayes and Cai (2007) (see also, Shkoler and Kimura, 2020), to ensure that the estimator of the covariance matrix of the parameter estimates is not biased and inconsistent under violation of heteroscedasticity. All the results are depicted in Table 2 and Figures 2–6.

**TABLE 2:** Moderated-mediation results (perception of organizational politics [POP] as the moderator).

Path (IV)		MV		DV	*b*	*SE*	*Sig.*
Abusive leadership	→	**LMX**	(*R*^2^ = 0.44) _(common/repeating path)_				
Abusive leadership					–0.45	0.06	0.000
Organizational politics					–0.36	0.04	0.000
**INT**_(Abusive × Org. Politics)_					0.08	0.03	0.007
Abusive leadership	→	LMX	→	**CWB** (*R*^2^ = 0.27)			
Abusive leadership					0.25	0.06	0.000
LMX					–0.30	0.03	0.000
Organizational politics					0.06	0.03	0.012
**INT**_(Abusive × Org. Politics)_					0.11	0.03	0.000
Abusive leadership	→	LMX	→	**OCB** (*R*^2^ = 0.12)			
Abusive leadership					0.07	0.05	0.126
LMX					0.27	0.04	0.000
Organizational politics					–0.01	0.03	0.756
**INT**_(Abusive × Org. Politics)_					–0.11	0.04	0.002
Abusive leadership	→	LMX	→	**Turnover** (*R*^2^ = 0.28)			
Abusive leadership					0.04	0.09	0.661
LMX					–0.56	0.08	0.000
Organizational politics					0.43	0.08	0.000
**INT**_(Abusive × Org. Politics)_					0.12	0.06	0.039

Bold facilitates readability. IV, independent variable (predictor); MV, mediator variable; DV, dependent variable (outcome); INT, interaction effect; Org., organizational; LMX, leader–member exchange; CWB, counterproductive work behavior; OCB, organizational citizenship behavior.

Table 2 and Figure 2 indicate that there are four statistically significant interaction effects, and, as such, it is evident in the data that POP moderates the relationship between: (1) abusive leadership and LMX; (2) LMX and CWB; (3) LMX and OCB; and (4) LMX and TIs. The significant interaction effects are graphically depicted in Figures 3–6. Moreover, from further analyses, it is evident that LMX is indeed a *partial* mediator of the above-mentioned links: (a) Abusive Leadership→LMX→CWB (*p* = 0.008); (b) Abusive Leadership→LMX→OCB (*p* = 0.000); and (C) Abusive Leadership→LMX→TIs (*p* = 0.026). Hypothesis 5 was therefore fully supported. Also, pairwise contrasts between conditional indirect effects (i.e., moderated-mediation effects) indicate that the mediation effects, at the different values of the moderator, significantly differ from one another (i.e., indirect effect at –1*SD* of POP is significantly different from the indirect effect at 0*SD* and + 1*SD*, and the two latter indirect effects differ significantly as well).

As can be seen in Figure 3, the negative relationship between abusive leadership and LMX *diminishes* the higher the perception of POP are. In other words, as POP increases, the negative association between abusive leadership and LMX *weakens*.

As can be seen in Figure 4, the relationship between LMX and CWB changes dramatically based on the level of POP, so that for *low* POP the association is *negative*, but for *high* POP the association is *positive*, meaning an increase in POP weakens the negative link between LMX and CWB. The polarization depicted in Figure 4 is surprising, and will be addressed further in the discussion.

As can be seen in Figure 5, the positive relationship between LMX and OCB diminishes the higher the POP are. In other words, as POP increase, the positive association between LMX and OCB weakens.

As can be seen in Figure 6, the negative relationship between LMX and turnover intentions (TIs) diminishes the higher the POP are. In other words, as POP increase, the negative association between LMX and TIs weakens. The findings support Hypothesis H6.

Finally, to test the conditional indirect effect, full analyses are presented in Table 3.

**TABLE 3:** Conditional indirect effects analyses (LMX as mediator; perception of organizational politics [POP] as moderator).

Path/organizational politics level	Effect	*SE*	LL_95%_	UL_95%_	*p* < 0.05
**Abusive leadership → LMX → CWB_(abusive leadership on CWB through LMX)_**					
*LOW* (–1 SD) organizational politics	0.07	0.02	0.03	0.12	Yes
*MEAN* (0 SD) organizational politics	0.02	0.01	–0.02	0.04	No
*HIGH* (+ 1 SD) organizational politics	–0.04	0.02	–0.02	–0.01	Yes
**Abusive leadership → LMX → OCB_(abusive leadership on OCB through LMX)_**					
*LOW* (–1 SD) organizational politics	–0.19	0.04	–0.29	–0.12	Yes
*MEAN* (0 SD) organizational politics	–0.12	0.03	–0.18	–0.08	Yes
*HIGH* (+ 1 SD) organizational politics	–0.06	0.02	–0.11	–0.03	Yes
**Abusive leadership → LMX → turnover intentions_(abusive leadership on TI through LMX)_**					
*LOW* (–1 SD) organizational politics	0.38	0.08	0.20	0.51	Yes
*MEAN* (0 SD) organizational politics	0.25	0.05	0.16	0.36	Yes
*HIGH* (+ 1 SD) organizational politics	0.18	0.04	0.09	0.27	Yes

Bold facilitates readability. LMX, leader–member exchange; CWB, counterproductive work behaviors; OCB, organizational citizenship behaviors; TI, turnover intentions; Effect, indirect effect size estimator. The number 95% = confidence level. LL, lower limit of 95% confidence interval (CI). UL, upper limit of 95% CI.

It can be seen in Table 3 that most of the conditional mediation effects are statistically significant, indicating *partial* mediation. The *only* instance in which the aforementioned indirect effect is non-significant is in the path Abusive Leadership→LMX→CWB (under a “*mean* organizational politics” condition).

## Discussion

The negative impact of abusive leadership has been well documented in the literature. The current paper focuses on the more complex interactions with two additional related variables. More specifically, it shed light on the associations between abusive leadership and organizational outcomes (OCB, CWB, and TI), as mediated by LMX and moderated by POP.

The results render almost full support to the study hypotheses. The findings indicate that: (1) abusive leadership damages LMX relations; (2) LMX is a partial mediator between abusive leadership and organizational outcomes; and (3) POP moderates this mediation.

The moderating effect of LMX on the relationships between abusive leadership and organizational outcomes has been suggested in the literature, and has gained empirical support in some studies (Xu et al., 2012, 2015; Pan and Lin, 2018). The results of the present study reaffirm the moderation effect but suggest that this moderation is partial, suggesting that abusive leadership’s negative impact is prominent enough to go beyond the moderating effect of LMX, and also has a direct negative effect on the employee.

Perhaps the most innovative finding of this study pertains to the role of POP in moderating the effects of abusive leadership and LMX on employee behavior.

Previous research has shown that the relations between abusive leadership and employees’ negative and resentful behavior can be moderated by other variables such as psychological ownership (Islam et al., 2022), Islamic work ethics and learning goal orientation (Islam et al., 2021a) and future role orientation (Islam et al., 2021b). These studies demonstrate that the negative impact of abusive supervision can be buffered by certain moderating variables. The present study suggests a more complex moderating effect. According to the present findings, under high POP, the negative impact of the immediate supervisor on employee’s negative work-related behavior is reduced.

We suggested earlier that while POP represents the perception of the organizational political environment, LMX and abusive leadership focus on the perception of one’s superior. When POP is high it tends to suppress the impact of the immediate superior on the employee’s, negative approach toward the organization. POP does not directly change the employee’s perception of the superior or the perception of the dyadic relationship between them, but the impact of these perceptions on the employee’s attitudes and behavior is reduced. There is greater concern about the undesirable political climate, while the concerns about the superior’s behavior and the quality of the relations with him lose some of their relevance to one’s attitudes and behavior.

The finding with respect to the link between LMX and CWB was more extreme and surprising, suggesting that under high POP, LMX becomes positively rather than negatively related to CWB. It may be speculated that when the superior–subordinate dyadic relations are good, the employee may feel that the superior will be relatively tolerant to undesirable behavior, which may be inspired by the employee’s frustration with the high political climate. The employee may even assume that their superior is also frustrated with the high political climate, and might have some understanding of this form of frustration-driven behavior. Clearly, this line of reasoning is highly speculative. Further understanding of this finding may be obtained in the future through different investigative techniques such as open interviews.

### Limitations and suggestions for future research

First, ours is a cross-sectional research design, and although cross-lagged data are not always warranted (e.g., Spector, 2019), they often offer superior statistical inference (e.g., Stinglhamber and Vandenberghe, 2003; Vandenberghe et al., 2011). As such, we recommend testing a similar model in a longitudinal perspective.

Second, this paper focused on two important exchange foci in the organization: employee–supervisor and employee–organization (Cole et al., 2002). However, it would also be interesting to explore the effects of abusive leadership and organizational politics on the third focus of these exchanges—among group/team members (Cole et al., 2002). Because humans often learn by watching others (their behaviors, rewards, and punishments) to direct and shape their own behaviors (Bandura and Walters, 1977; Bandura, 2018), it is safe to assume that abusive leadership creates collateral damage.

Third, the current research model attempted to shed light on contextual factors in relation to abusive leadership, LMX and organizational outcomes. This might limit our understanding of the investigated phenomena. As such, we recommend future research to include individual differences as well (e.g., personality dispositions), in the aim of drawing a broader, more holistic, and more reality-representative picture, as was suggested by eminent scholars (e.g., Staw and Cohen-Charash, 2005; Judge and Zapata, 2015).

Finally, this study presents cross-sectional analyses that would greatly benefit from replications in various cultural settings. This is important especially for cross-cultural management (e.g., Thomas and Peterson, 2016). The fact that each country is unique (Hofstede, 1980, 1991) invokes interest in replicating the current research (and others as well) in different countries/cultures, largely increasing external and construct validities of the results. Replications should *not* be discouraged, as they provide the surest method for stability and credibility of any research model. This notion coincides with the recommendation of eminent scholars arguing that the ultimate test for validity of findings is their recurrence in numerous replications (James et al., 1982).

Finally, the most immediate practical implication of the present results to the management of organizations pertains to educating management about the importance of organizational politics in affecting employees’ behavioral inclinations. This study points at the possibility that beyond the direct effects on employee’s behavior, POP might suppress or inhibit the impact of the immediate supervisory level on the employee. The present study specifically examined two aspects of the superior–subordinate interface. The potential role of POP in suppressing other aspects of this interface is yet to be explored. Organizational top management should become aware of the potential impact of organizational politics as demonstrated in this study. Such increased awareness may encourage management to make a greater effort to prevent organizational politics from playing an important role in a given organizational climate.

## Data availability statement

The dataset(s) generated during and/or analyzed during the current study are not publicly available due to discretion and anonymity considerations, but are available from the corresponding author on reasonable request.

## Ethics statement

Ethical review and approval was not required for the study on human participants in accordance with the local legislation and institutional requirements. At the beginning of each questionnaire, the general goal of the research was explained. Informed consent was obtained individually from all participants included in the study. We ensured maximum anonymity and discretion of the results, and also ensured the subjects knew they could leave and stop their participation at any time they choose to do so.

## Author contributions

All authors listed have made a substantial, direct, and intellectual contribution to the work, and approved it for publication.

## Footnotes

1
https://www.prolific.co/

## References

[B1] Adams J. S. (1965). Inequity in social exchange. *Adv. Exp. Soc. Psychol.* 2 267–299. 10.1016/S0065-2601(08)60108-2

[B2] Aharoni-Goldberg S. Tziner A. Barnett D. (2019). Repercussions of incivility and hostile expressions in academia: A legal perspective. *Ind. Organ. Psychol.* 12 385–390. 10.1017/iop.2019.72

[B3] Aubé C. Rousseau V. Mama C. Morin E. M. (2009). Counterproductive behaviors and psychological well-being: The moderating effect of task interdependence. *J. Bus. Psychol.* 24 351–361. 10.1007/s10869-009-9113-5

[B4] Bandura A. (2018). “Albert Bandura and social learning theory,” in *Learning theories for early years practice*, ed. MacBlain S. (Thousand Oaks, CA: Sage), 63–65.

[B5] Bandura A. Walters R. H. (1977). *Social learning theory*, Vol. 1. Hoboken, NJ: Prentice-Hall.

[B6] Barrick M. Mount M. (1993). Autonomy as a moderator of the relationship between the Big Five personality dimensions and job performance. *J. Appl. Psychol.* 78 111–118.

[B7] Bennett R. J. Robinson S. L. (2000). Development of a measure of workplace deviance. *J. Appl. Psychol.* 85 349–360. 10.1037/0021-9010.85.3.349 10900810

[B8] Blau P. M. (1964). *Exchange and power in social life.* New York, NY: John Wiley & Sons.

[B9] Bokek-Cohen Y. Shkoler O. Meiri E. (2022). The unique practices of workplace bullying in academe and their prevalence: An exploratory study. *Curr. Psychol.* 1–20.

[B10] Bowling N. Michel J. (2011). Why do you treat me badly? The role of attributions regarding the cause of abuse in subordinates responses to abusive supervision. *Work Stress* 25 309–320. 10.1080/02678373.2011.634281

[B11] Breevaart K. Bakker A. B. Demerouti E. Van Den Heuvel M. (2015). Leader-member exchange, work engagement, and job performance. *J. Manage. Psychol.* 30 754–770. 10.1108/JMP-03-2013-0088

[B12] Byrne B. M. (2010). *Structural equation modelling with AMOS: Basic concepts, applications, and programming*, 2nd Edn. Milton Park: Taylor & Francis Group.

[B13] Cammann C. Fichman M. Jenkins D. Klesh J. (1979). *The michigan organizational assessment questionnaire [Unpublished manuscript].* Ann Arbor, MI: University of Michigan.

[B14] Chen H. Richard O. C. Boncoeur O. D. Ford D. L. Jr. (2020). Work engagement, emotional exhaustion, and counterproductive work behavior. *J. Bus. Res.* 114 30–41. 10.1016/j.jbusres.2020.03.025

[B15] Chen P. Y. Spector P. E. (1992). Relationships of work stressors with aggression, withdrawal, theft and substance use: An exploratory study. *J. Occup. Organ. Psychol.* 65 177–184. 10.1111/j.2044-8325.1992.tb00495.x

[B16] Chernyak-Hai L. Tziner A. (2014). Relationships between counterproductive work behavior, perceived justice and climate, occupational status, and leader-member exchange. *J. Work Organ. Psychol.* 30 1–12.

[B17] Cohen-Charash Y. Mueller J. S. (2007). Does perceived unfairness exacerbate or mitigate interpersonal counterproductive work behaviors related to envy? *J. Appl. Psychol.* 92 666–680. 10.1037/0021-9010.92.3.666 17484549

[B18] Cole M. S. Schaninger W. S. Jr. Harris S. G. (2002). The workplace social exchange network: A multilevel, conceptual examination. *Group Organ. Manage.* 27 142–167. 10.1177/1059601102027001008

[B19] Cropanzano R. Byrne Z. S. Bobocel D. R. Rupp D. E. (2001). Moral virtues, fairness heuristics, social entities, and other denizens of organizational justice. *J. Vocat. Behav.* 58 164–209. 10.1006/jvbe.2001.1791

[B20] Daileyl R. C. Kirk D. J. (1992). Distributive and procedural justice as antecedents of job dissatisfaction and intent to turnover. *Hum. Relat.* 45 305–317. 10.1177/001872679204500306

[B21] Dansereau F. Jr. Graen G. Haga W. J. (1975). A vertical dyad linkage approach to leadership within formal organizations: A longitudinal investigation of the role making process. *Organ. Behav. Hum. Perform.* 13 46–78. 10.1016/0030-5073(75)90005-7

[B22] Drory A. Romm T. (1990). The definition of organizational politics—a review. *Hum. Relat.* 43:1154.

[B23] Duffy M. K. Ganster D. C. Pagon M. (2002). Social undermining in the workplace. *Acad. Manage. J.* 45 331–351. 10.5465/3069350 3069350

[B24] Durrani A. B. (2014). Understanding the relationship between organizational politics and leadership styles. *J. Bus. Manage.* 16 62–67.

[B25] Ferris G. R. Adams G. Kolodinsky R. W. Hochwarter W. A. Ammeter A. P. (2002). “Perceptions of organizational politics: Theory and research directions,” in *Research in multi-level issues: The many faces of multi-level issues*, Vol. 1 eds Yammarino F. J. Dansereau F. (Amsterdam, NL: Elsevier Science), 179–254.

[B26] Ferris G. R. Frink D. D. Galang M. C. Zhou J. Kacmar K. M. Howard J. L. (1996). Perceptions of organizational politics: Predictors, stress-related implications, and outcomes. *Hum. Relat.* 49 233–266.

[B27] Festinger L. (1954). A theory of social comparison processes. *Hum. Relat.* 7 117–140. 10.1177/001872675400700202

[B28] Festinger L. (1957). *A theory of cognitive dissonance.* Evanston, IL: Row, Peterson.

[B29] Gouldner A. W. (1960). The norm of reciprocity: A preliminary statement. *Am. Sociol. Rev.* 25 161–178. 10.2307/2092623

[B30] Graen G. Cashman J. F. (1975). “A role-making model of leadership in formal organizations: A developmental approach,” in *Leadership frontiers*, eds Hung J. G. Larsen L. L. (Kent, OH: Kent State University Press), 143–165.

[B31] Graen G. Uhl-Bien M. (1995). Relationship-based approach to leadership: Development of leader-member exchange (LMX) theory of leadership over 25 years: Applying a multi-level multi-domain perspective. *Leadersh. Q.* 6 219–247. 10.1016/1048-9843(95)90036-5

[B32] Greenberg J. Scott K. S. (1996). “Why do workers bite the hands that feed them? Employee theft as a social exchange process,” in *Research in organizational behavior*, Vol. 18 eds Staw B. M. Cummings L. L. (Greenwich, CT: JAI Press), 111–156.

[B33] Griffeth R. W. Hom P. W. Gaertner S. (2000). A meta-analysis of antecedents and correlates of employee turnover: Update, moderator tests, and research implications for the next millennium. *J. Manage.* 26 463–488. 10.1177/014920630002600305

[B34] Gruys M. L. Sackett P. R. (2003). Investigating the dimensionality of counterproductive work behavior. *Int. J. Sel. Assess.* 11 30–42. 10.1111/1468-2389.00224

[B35] Harris K. J. Harvey P. Kacmar K. M. (2011). Abusive supervisory reactions to coworker relationship conflict. *Leadersh. Q.* 22 1010–1023. 10.1016/j.leaqua.2011.07.020

[B36] Hayes A. F. Cai L. (2007). Using heteroskedasticity-consistent standard error estimators in OLS regression: An introduction and software implementation. *Behav. Res. Methods* 39 709–722. 10.3758/BF03192961 18183883

[B37] Ho V. T. (2012). Interpersonal counterproductive work behaviors: Distinguishing between person-focused versus task-focused behaviors and their antecedents. *J. Bus. Psychol.* 27 467–482. 10.1007/s10869-012-9256-7

[B38] Hobfoll S. E. (1989). Conservation of resources: A new attempt at conceptualizing stress. *Am. Psychol.* 44 513–524. 10.1037//0003-066x.44.3.513 2648906

[B39] Hobfoll S. E. (2011). Conservation of resource caravans and engaged settings. *J. Occup. Organ. Psychol.* 84 116–122. 10.1111/j.2044-8325.2010.02016.x

[B40] Hochwarter W. A. Perrewé P. L. Ferris G. R. Guercio R. (1999). Commitment as an antidote to the tension and turnover consequences of organizational politics. *J. Vocat. Behav.* 55 277–297.

[B41] Hofstede G. (1980). *Culture’s consequences: International differences in work-related values.* Thousand Oaks, CA: Sage.

[B42] Hofstede G. (1991). *Cultures and organization: Software of the mind.* New York, NY: McGraw-Hill.

[B43] Hom P. W. Lee T. W. Shaw J. D. Hausknecht J. P. (2017). One hundred years of employee turnover theory and research. *J. Appl. Psychol.* 102 530–545. 10.1037/apl0000103 28125259

[B44] Hom P. W. Mitchell T. R. Lee T. W. Griffeth R. W. (2012). Reviewing employee turnover: Focusing on proximal withdrawal states and an expanded criterion. *Psychol. Bull.* 138 831–858. 10.1037/a0027983 22925138

[B45] Homans G. C. (1958). Social behavior as exchange. *Am. J. Sociol.* 63 597–606. 10.1086/222355

[B46] Ilies R. Nahrgang J. D. Morgeson F. P. (2007). Leader-member exchange and citizenship behaviors: A meta-analysis. *J. Appl. Psychol.* 92 269–277. 10.1037/0021-9010.92.1.269 17227168

[B47] Islam T. Ahmad S. Kaleem A. Mahmood K. (2021a). Abusive supervision and knowledge sharing: Moderating roles of Islamic work ethic and learning goal orientation. *Manage. Decis.* 59 205–222. 10.1108/MD-08-2019-1069

[B48] Islam T. Ahmed I. Usman A. Ali M. (2021b). Abusive supervision and knowledge hiding: The moderating roles of future orientation and Islamic work ethics. *Manage. Res. Rev.* 44 1565–1582. 10.1108/MRR-06-2020-0348

[B49] Islam T. Hussain M. (2022). How consumer uncertainty intervene country of origin image and consumer purchase intention? The moderating role of brand image. *Int. J. Emerg. Marke.* (ahead-of-print). 10.1108/IJOEM-08-2021-1194

[B50] Islam T. Asif A. Jamil S. Ali H. F. (2022). How abusive supervision affect knowledge hiding? The mediating role of employee silence and moderating role of psychological ownership. *VINE J. Inform. Knowl. Manage. Syst.* (ahead-of-print). 10.1108/VJIKMS-11-2021-0274

[B51] Jain A. K. Srivastava S. Cooper C. (2021). A study on the relationship of abusive supervision and fear-based silence in India the mediating role of dimensions of emotional intelligence. *Curr. Psychol.* (ahead-of-print). 10.1007/s12144-021-01912-3

[B52] James L. R. Mulaik S. A. Brett J. M. (1982). *Conditions for confirmatory analysis and causal inference.* Thousand Oaks, CA: Sage.

[B53] Judge T. A. Zapata C. P. (2015). The person–situation debate revisited: Effect of situation strength and trait activation on the validity of the Big Five personality traits in predicting job performance. *Acad. Manage. J.* 58 1149–1179. 10.5465/amj.2010.0837

[B54] Kacmar K. M. Carlson D. S. (1997). Further validation of the perceptions of politics scale (POPS): A multiple sample investigation. *J. Manage.* 23 627–658. 10.1177/014920639702300502

[B55] Kacmar K. M. Ferris G. R. (1991). Perceptions of organizational politics scale (POPS): Development and construct validation. *Educ. Psychol. Measur.* 51 193–205. 10.1177/0013164491511019

[B56] Kelloway E. K. Francis L. Prosser M. Cameron J. E. (2010). Counterproductive work behavior as protest. *Hum. Resour. Manage. Rev.* 20 18–25. 10.1016/j.hrmr.2009.03.014

[B57] Kish-Gephart J. J. Detert J. R. Treviño L. K. Edmondson A. C. (2009). Silenced by fear: The nature, sources, and consequences of fear at work. *Res. Organ. Behav.* 29 163–193. 10.1016/j.riob.2009.07.002

[B58] Korman B. A. Tröster C. Giessner S. R. (2021). The consequences of incongruent abusive supervision: Anticipation of social exclusion, shame, and turnover intentions. *J. Leadersh. Organ. Stud.* 28 306–321. 10.1177/15480518211005463

[B59] Lam L. W. Xu A. J. (2019). Power imbalance and employee silence: The role of abusive leadership, power distance orientation, and perceived organisational politics. *Appl. Psychol.* 68 513–546. 10.1111/apps.12170

[B60] Lebrón M. Tabak F. Shkoler O. Rabenu E. (2018). Counterproductive work behaviors toward organization and leader-member exchange: The mediating roles of emotional exhaustion and work engagement. *Organ. Manage. J.* 15 159–173. 10.1080/15416518.2018.1528857

[B61] Lee A. Gerbasi A. Schwarz G. Newman A. (2019). Leader–member exchange social comparisons and follower outcomes: The roles of felt obligation and psychological entitlement. *J. Occup. Organ. Psychol.* 92 593–617. 10.1111/joop.12245

[B62] Lee T. W. Mitchell T. R. (1994). An alternative approach: The unfolding model of voluntary employee turnover. *Acad. Manage. Rev.* 19 51–89. 10.5465/amr.1994.9410122008

[B63] Lee T. W. Hom P. Eberly M. Li J. J. (2018). Managing employee retention and turnover with 21st century ideas. *Organ. Dyn.* 47 88–98. 10.1016/j.orgdyn.2017.08.004

[B64] Lian H. Ferris D. L. Morrison R. Brown D. J. (2014). Blame it on the supervisor or the subordinate? Reciprocal relations between abusive supervision and organizational deviance. *J. Appl. Psychol.* 99 651–664. 10.1037/a0035498 24377392

[B65] Liao Z. Lee H. W. Johnson R. E. Song Z. Liu Y. (2021). Seeing from a short-term perspective: When and why daily abusive supervisor behavior yields functional and dysfunctional consequences. *J. Appl. Psychol.* 106 377–398. 10.1037/apl0000508 32352822

[B66] Lind E. A. Tyler T. R. (1988). *The social psychology of procedural justice.* New York, NY: Plenum.

[B67] Mackey J. D. Frieder R. E. Brees J. R. Martinko M. J. (2017). Abusive supervision: A meta-analysis and empirical review. *J. Manage.* 43 1940–1965. 10.1177/0149206315573997

[B68] Martin R. Guillaume Y. Thomas G. Lee A. Epitropaki O. (2016). Leader-member exchange (LMX) and performance: A meta-analytic review. *Pers. Psychol.* 69 67–121. 10.1111/peps.12100

[B69] Martinko M. J. Harvey P. Brees J. R. Mackey J. (2013). A review of abusive supervision research. *J. Organ. Behav.* 34 S120–S137. 10.1002/job.1888

[B70] Matta F. K. Van Dyne L. (2020). Understanding the disparate behavioral consequences of LMX differentiation: The role of social comparison emotions. *Acad. Manage. Rev.* 45 154–180. 10.5465/amr.2016.0264

[B71] Mauss M. (1967). *The gift: Forms and functions of exchange in archaic societies.* Arizona: Norton.

[B72] Mawritz M. B. Dust S. B. Resick C. J. (2014). Hostile climate, abusive supervision, and employee coping: Does conscientiousness matter? *J. Appl. Psychol.* 99 737–747. 10.1037/a0035863 24512026

[B73] McLarty B. D. Muldoon J. Quade M. King R. A. (2021). Your boss is the problem and solution: How supervisor-induced hindrance stressors and LMX influence employee job neglect and subsequent performance. *J. Bus. Res.* 130 308–317. 10.1016/j.jbusres.2021.03.032

[B74] Mitchell M. S. Ambrose M. L. (2007). Abusive supervision and workplace deviance and the moderating effects of negative reciprocity beliefs. *J. Appl. Psychol.* 92 1159–1168. 10.1037/0021-9010.92.4.1159 17638473

[B75] Naseer S. Raja U. Syed F. Donia M. B. Darr W. (2016). Perils of being close to a bad leader in a bad environment: Exploring the combined effects of despotic leadership, leader member exchange, and perceived organizational politics on behaviors. *Leadersh. Q.* 27 14–33. 10.1016/j.leaqua.2015.09.005

[B76] Naseer S. Bouckenooghe D. Syed F. Khan A. K. Qazi S. (2020). The malevolent side of organizational identification: Unraveling the impact of psychological entitlement and manipulative personality on unethical work behaviors. *J. Bus. Psychol*. 35, 333–346. 10.1007/s10869-019-09623-0

[B77] Newton C. Perlow R. (2021). The role of leader-member exchange relations and individual differences on counterproductive work behavior. *Psychol. Rep.* (ahead-of-print). 10.1177/0033294121989298 33517838

[B78] Ng T. W. Feldman D. C. (2012). Employee voice behavior: A meta-analytic test of the conservation of resources framework. *J. Organ. Behav.* 33 216–234. 10.1002/job.754

[B79] Oliver R. (1974). Expectancy theory predictions of salesmen’s performance. *J. Marke. Res.* 11 243–253. 10.1177/002224377401100302

[B80] Organ D. W. (1988). *Organizational citizenship behavior: The good soldier syndrome*. Lexington, MA: Lexington.

[B81] Organ D. W. Podsakoff P. M. MacKenzie S. B. (2006). *Organizational citizenship behavior: Its nature, antecedents and consequences.* Beverly Hills, CA: Sage.

[B82] Palanski M. Avey J. B. Jiraporn N. (2014). The effects of ethical leadership and abusive supervision on job search behaviors in the turnover process. *J. Bus. Ethics* 121 135–146. 10.1007/s10551-013-1690-6

[B83] Pan S.-Y. Lin K. (2018). Who suffers when supervisors are unhappy? The roles of leader–member exchange and abusive supervision. *J. Bus. Ethics* 151 799–811.

[B84] Park T.-Y. Shaw J. D. (2013). Turnover rates and organizational performance: A meta-analysis. *J. Appl. Psychol.* 98 268–309. 10.1037/a0030723 23244224

[B85] Podsakoff P. M. MacKenzie S. B. Lee J. Y. Podsakoff N. P. (2003). Common method biases in behavioral research: A critical review of the literature and recommended remedies. *J. Appl. Psychol.* 88 879–903. 10.1037/0021-9010.88.5.879 14516251

[B86] Porter L. W. Allen R. W. Angle H. L. (1981). “The politics of upward influence in organizations,” in *Research in organizational behavior*, Vol. 3 eds Cummings L. L. Staw B. M. (Greenview, CT: JAI Press), 109–149.

[B87] Rice D. B. Taylor R. Forrester J. K. (2020). The unwelcoming experience of abusive supervision and the impact of leader characteristics: Turning employees into poor organizational citizens and future quitters. *Eur. J. Work Org. Psychol*. 29, 601–618. 10.1080/1359432X.2020.1737521

[B88] Robinson S. Bennett R. (1995). A typology of deviant workplace behaviors: A multi- dimensional ranging study. *Acad. Manage. J.* 38 555–572. 10.2307/256693

[B89] Sackett P. R. DeVore C. J. (2001). “Counterproductive behaviors at work,” in *Handbook of industrial, work, & organizational psychology*, Vol. 1 eds Anderson N. Ones D. S. Sinangil H. K. Viswesvaran C. (Thousand Oaks, CA: Sage), 145–164. 10.3390/ijerph18179416

[B90] Salton Meyer E. Ein-Dor T. (2021). “Psychological and organizational antecedents and consequences of abusive supervision in Israel: Review and research,” in *Asian perspectives on workplace bullying and harassment*, eds D’Cruz P. Noronha E. Mendonca A. (Berlin: Springer), 211–244. 10.1007/978-981-16-2362-2_8

[B91] Shapira-Lishchinsky O. (2005). *Organizational ethics as predictors of teachers’ work withdrawal behaviors: Absence, lateness and tendency to leave [Unpublished doctoral dissertation].* Israel: University of Haifa.

[B92] Sharif M. Scandura T. A. (2017). A little give and take: The exchange of culture in leader-member exchange. *Acad. Manage. Proc.* 2017:14619. 10.5465/AMBPP.2017.14619abstract

[B93] Shkoler O. Kimura T. (2020). How does work motivation impact employees investment at work and their job engagement? A moderated-moderation perspective through an international lens. *Front. Psychol.* 11:38. 10.3389/fpsyg.2020.00038 32153446 PMC7046595

[B94] Shkoler O. Tziner A. (2017). The mediating and moderating role of burnout and emotional intelligence in the relationship between organizational justice and work misbehavior. *J. Work Organ. Psychol.* 33 157–164. 10.1016/j.rpto.2017.05.002

[B95] Shkoler O. Tziner A. (2020). Leadership styles as predictors of work attitudes: A moderated-mediation link. *Amfiteatru Econom.* 22 164–178. 10.24818/EA/2020/53/164

[B96] Shkoler O. Rabenu E. Tabak F. Lebrón M. J. (2019). Leader- and team-member exchanges and their relationships with organizational and interpersonal counterproductive work behaviors: Moderation by envy and group size in Israel and USA. *J. Work Organ. Psychol.* 35 145–156. 10.5093/jwop2019a19

[B97] Shkoler O. Tziner A. Vasiliu C. Ghinea C. N. (2021). A moderated-mediation analysis of organizational justice and leader-member exchange: Cross-validation with three sub-samples. *Front. Psychol.* 12:616476. 10.3389/fpsyg.2021.616476 34248733 PMC8267068

[B98] Shore L. M. Randel A. E. Chung B. G. Dean M. A. Ehrhart K. H. Singh G. (2011). Inclusion and diversity in work groups: A review and model for future research. *J. Manage.* 37 1262–1289. 10.1177/0149206310385943

[B99] Shoss M. Eisenberger R. Restubog S. Zagenczyk T. (2013). Blaming the organization for abusive supervision: The roles of perceived organizational support and supervisor’s organizational embodiment. *J. Appl. Psychol.* 98 158–168. 10.1037/a0030687 23205496

[B100] Skarlicki D. P. Folger R. (1997). Retaliation in the workplace: The roles of distributive, procedural, and interactional justice. *J. Appl. Psychol.* 82 434–443. 10.1037/0021-9010.82.3.434

[B101] Spector P. E. (2019). Do not cross me: Optimizing the use of cross-sectional designs. *J. Bus. Psychol.* 34 125–137. 10.1007/s10869-018-09613-8

[B102] Spector P. E. Bauer J. A. Fox S. (2010). Measurement artifacts in the assessment of counterproductive work behavior and organizational citizenship behavior: Do we know what we think we know? *J. Appl. Psychol.* 95 781–790. 10.1037/a0019477 20604597

[B103] Spector P. E. Fox S. Penney L. M. Bruursema K. Goh A. Kessler S. (2006). The dimensionality of counterproductivity: Are all counterproductive behaviors created equal? *J. Vocat. Behav.* 68 446–460. 10.1016/j.jvb.2005.10.005

[B104] Staw B. M. Cohen-Charash Y. (2005). The dispositional approach to job satisfaction: More than a mirage, but not yet an oasis. *J. Organ. Behav.* 26 59–78. 10.1002/job.299

[B105] Steffens N. K. Yang J. Jetten J. Haslam S. A. Lipponen J. (2018). The unfolding impact of leader identity entrepreneurship on burnout, work engagement, and turnover intentions. *J. Occup. Health Psychol.* 23 373–387. 10.1037/ocp0000090 28749160

[B106] Stinglhamber F. Vandenberghe C. (2003). Organizations and supervisors as sources of support and targets of commitment: A longitudinal study. *J. Organ. Behav.* 24 251–270. 10.1002/job.192

[B107] Teng C. C. Lu A. C. C. Huang Z. Y. Fang C. H. (2020). Ethical work climate, organizational identification, leader-member-exchange (LMX) and organizational citizenship behavior (OCB): A study of three-star hotels in Taiwan. *Int. J Contemp. Hosp. Manage.* 32 212–229. 10.1108/IJCHM-07-2018-0563

[B108] Tepper B. J. (2000). Consequences of abusive supervision. *Acad. Manage. J.* 43 178–190. 10.2307/1556375

[B109] Thomas D. C. Peterson M. F. (2016). *Cross-cultural management: Essential concepts.* Thousand Oaks, CA: Sage Publications.

[B110] Thompson M. J. Carlson D. S. Hackney K. Vogel R. M. (2021). Vicarious abusive supervision and turnover in expectant working mothers: Does financial dependency trigger emotional disconnect? *J. Organ. Behav.* 43 448–464.

[B111] Tröster C. Van Quaquebeke N. (2021). When victims help their abusive supervisors: The role of LMX, self-blame, and guilt. *Acad. Manage. J.* 64 1793–1815. 10.5465/amj.2019.0559

[B112] Tyler T. R. Lind E. A. (1992). “A relational model of authority in groups,” in *Advances in experimental social psychology*, Vol. 25 ed. Zanna M. (Cambridge, MA: Academic Press), 115–191.

[B113] Tziner A. Shkoler O. Fein E. C. (2020). Examining the effects of cultural value orientations, emotional intelligence, and motivational orientations: How do LMX mediation and gender-based moderation make a difference? *Frontiers in Psychology* 11:502903. 10.3389/fpsyg.2020.502903 33192756 PMC7643027

[B114] Valle M. P. Perrewé P. L. (2000). Do politics perceptions relate to political behaviors? *Human Relations* 53 359–386.

[B115] Vandenberghe C. Panaccio A. Bentein K. Mignonac K. Roussel P. (2011). Assessing longitudinal change of and dynamic relationships among role stressors, job attitudes, turnover intention, and well-being in neophyte newcomers. *J. Organ. Behav.* 32 652–671. 10.1002/job.732

[B116] Vardi Y. Weitz E. (2016). *Misbehavior in organizations: A dynamic approach*, 2nd Edn. London: Routledge. 10.4324/9781315732565

[B117] Vigoda E. (2000). Internal politics in public administration system: An empirical examination of its relationship with job congruence, organizational citizenship behavior, and in-role performance. *Public Pers. Manage.* 29 185–210. 10.1177/009102600002900203

[B118] Vigoda-Gadot E. Drory A. (eds) (2016). *Handbook of organizational politics: Looking back and to the future*, 2nd Edn. Cheltenham: Edward Elgar.

[B119] Vroom V. H. (1964). *Work and motivation.* New York, NY: Wiley.

[B120] Williams L. J. Anderson S. E. (1991). Job satisfaction and organizational commitment as predictors of organizational citizenship and in-role behaviors. *J. Manage.* 17 601–617. 10.1177/014920639101700305

[B121] Xu A. Loi R. Lam L. (2015). The bad boss takes it all: How abusive supervision and leader–member exchange interact to influence employee silence. *Leadersh. Q.* 26 763–774. 10.1016/j.leaqua.2015.03.002

[B122] Xu E. Huang X. Lam C. Miao Q. (2012). Abusive supervision and work behaviors: The mediating role of LMX. *J. Organ. Behav.* 33 531–543. 10.1002/job.768

[B123] Xu Q. Zhao Y. Xi M. Li F. (2020). Abusive supervision, high-performance work systems, and subordinate silence. *Pers. Rev.* 49 1637–1653. 10.1108/PR-01-2019-0029

[B124] Zhang C. Mayer D. M. Hwang E. (2018). More is less: Learning but not relaxing buffers deviance under job stressors. *J. Appl. Psychol.* 103 123–136. 10.1037/apl0000264 28933912

